# Prognostic stratification of glioblastoma patients by unsupervised clustering of morphology patterns on whole slide images furthering our disease understanding

**DOI:** 10.3389/fnins.2024.1304191

**Published:** 2024-05-20

**Authors:** Bhakti Baheti, Shubham Innani, MacLean Nasrallah, Spyridon Bakas

**Affiliations:** ^1^Division of Computational Pathology, Department of Pathology and Laboratory Medicine, Indiana University School of Medicine, Indianapolis, IN, United States; ^2^Center for Artificial Intelligence and Data Science for Integrated Diagnostics (AI2D) and Center for Biomedical Image Computing and Analytics (CBICA), University of Pennsylvania, Philadelphia, PA, United States; ^3^Department of Pathology and Laboratory Medicine, Perelman School of Medicine, University of Pennsylvania, Philadelphia, PA, United States; ^4^Department of Radiology, Perelman School of Medicine, University of Pennsylvania, Philadelphia, PA, United States; ^5^Department of Computer Science, Luddy School of Informatics, Computing, and Engineering, Indiana University, Indianapolis, IN, United States

**Keywords:** glioblastoma, morphology, survival, clustering, stratification, computational pathology

## Abstract

**Introduction:**

Glioblastoma (GBM) is a highly aggressive malignant tumor of the central nervous system that displays varying molecular and morphological profiles, leading to challenging prognostic assessments. Stratifying GBM patients according to overall survival (OS) from H&E-stained whole slide images (WSI) using advanced computational methods is challenging, but with direct clinical implications.

**Methods:**

This work is focusing on GBM (IDH-wildtype, CNS WHO Gr.4) cases, identified from the TCGA-GBM and TCGA-LGG collections after considering the 2021 WHO classification criteria. The proposed approach starts with patch extraction in each WSI, followed by comprehensive patch-level curation to discard artifactual content, i.e., glass reflections, pen markings, dust on the slide, and tissue tearing. Each patch is then computationally described as a feature vector defined by a pre-trained VGG16 convolutional neural network. Principal component analysis provides a feature representation of reduced dimensionality, further facilitating identification of distinct groups of morphology patterns, via unsupervised k-means clustering.

**Results:**

The optimal number of clusters, according to cluster reproducibility and separability, is automatically determined based on the rand index and silhouette coefficient, respectively. Our proposed approach achieved prognostic stratification accuracy of 83.33% on a multi-institutional independent unseen hold-out test set with sensitivity and specificity of 83.33%.

**Discussion:**

We hypothesize that the quantification of these clusters of morphology patterns, reflect the tumor's spatial heterogeneity and yield prognostic relevant information to distinguish between short and long survivors using a decision tree classifier. The interpretability analysis of the obtained results can contribute to furthering and quantifying our understanding of GBM and potentially improving our diagnostic and prognostic predictions.

## 1 Introduction

Glioblastoma (GBM) is the most common malignant adult brain tumor with poor prognosis and heterogeneous morphology and molecular profiles (Verhaak et al., 2010; Brennan et al., 2013; Sottoriva et al., 2013). Specifically, there have been no substantial improvements in patient prognosis since 2005, with the median overall survival (OS) of GBM patients after standard-of-care treatment being 15 months and four months otherwise (Baid et al., 2020). Although GBM is the most common malignant adult primary brain tumor, it is characterized as a “rare” disease based on its incidence rate (i.e., 3/100,000 people), which is substantially lower than the rare disease definition, i.e., < 10/100,000 people (Griggs et al., 2009). This makes it challenging to find large and diverse data for developing machine learning diagnostic and prognostic models for GBM patients. Histopathologic evaluation of GBM tissue sections cut onto glass slides has always been the routine front-line assessment to provide essential information for disease diagnosis, treatment, and management. Even though the evaluation of histopathology slides by expert pathologists remains the gold standard for disease diagnosis and prognosis, such visual assessment is subjective and subject to interobserver variability. Recent technological gains have led to the routine scanning of conventional glass slides to high-quality digitized tissue sections - also known as whole slide images (WSI). WSIs present tumor morphology in very detailed, gigapixel resolution, which is extremely useful in cancer studies, enabling computational analysis and remote assessment by experts. Automatic computational analysis of WSI is a rapidly expanding field in medical image analysis, which can alleviate pathologists' workloads and help reduce the chance of diagnostic errors (Janowczyk and Madabhushi, 2016; Aeffner et al., 2019; Niazi et al., 2019; Baheti et al., 2023c). The broader use of WSI has, in turn, resulted in substantial developments in computational analysis of histopathology imaging, particularly for gaining novel insights from population-based studies.

There are several studies on prognostic stratification of GBM patients from radiology imaging (Macyszyn et al., 2015; Rathore et al., 2018b; Bakas et al., 2020; Beig et al., 2021). Based on the findings of these studies, we note a limit on the performance of these prognostic predictions that could be potentially addressed by computational analysis of WSI. However, computational analysis of WSIs for prognostic stratification of GBM patients face three major challenges. Firstly, WSIs usually describe image files of large storage requirements (measured in several gigabytes for a single WSI), as well as with spatial resolution in the range of 100, 000^2^ pixels. This large size of WSIs at their native spatial resolution makes handling and processing the entire image computationally demanding, in terms of both memory footprint and processing power. The typical workaround involves the tiling of the entire image into smaller patches, which raise major adverse effects and considerations for effective processing (Reina et al., 2020). Secondly, the inherent heterogeneity of GBM tumors has been well-accepted (Sottoriva et al., 2013), with their histologic composition being of varying morphological structures. Therefore, relying on a single WSI of the tumor may not accurately represent the morphological landscape of the complete tumor, potentially leading to incomplete or biased assessments (i.e., sampling error). To address this issue, multiple tissue sections might be considered for an analysis overcoming this sampling error, as well as toward better capturing the heterogeneity of GBMs, albeit this causes an exacerbation of the computational requirements challenge. Thirdly, the computational analysis of WSIs in the literature can be categorized into two approaches: i) patch-based, and ii) WSI-based. Due to the aforementioned challenges associated with handling the entire WSI, patch-based methods, focusing on specific annotated regions of interests (ROIs) marked by pathologists (Barker et al., 2016; Zhu et al., 2016; Cheng et al., 2018; Mobadersany et al., 2018), have formed the typical convention in the computational pathology field. Beyond the task of prognostic stratification, the field of computational pathology is limited by the need for pixel-level annotations of WSIs that are essential for detection and segmentation tasks. Such annotations are of limited availability as they are tedious and time-consuming, even for the expert pathologist, and hence hinder the application of supervised machine learning methods that rely on labeled data for model training.

Weakly supervised methods for WSI classification have seen advancements, incorporating multiple instance learning (Kather et al., 2019; Baheti et al., 2023a,b,d; Innani et al., 2023). However, many current approaches in the computational analysis of histopathology images heavily depend on hand-crafted imaging features extracted from a limited set of manually-labeled WSI patches. This limitation hinders their ability to capture the heterogeneous morphology of GBM effectively (Zhu et al., 2017). Additionally, understanding the features driving computational predictive decisions is crucial for potential data-driven enhancements in human expert assessments. Our approach utilizes an unsupervised clustering to identify distinct morphological patterns across WSI. The features extracted through this unsupervised approach capture intrinsic characteristics of tissue patterns, contributing to a more profound comprehension of morphological variations and structures within GBM. Unlike supervised approaches that rely on existing knowledge, unsupervised approaches can uncover new insights and knowledge by allowing data to directly inform the decision-making process. Importantly, the unsupervised method avoids any potential unconscious bias introduced by manual annotations, ensuring a more objective analysis of GBM morphology. The outcome of our approach lay the groundwork for furthering our current knowledge and enabling us to leverage data-driven identified morphological patterns for an improved assessment and novel insights.

Even though many computational studies attempt to develop a prognostic stratification model for GBM, their focus has not been on furthering our disease understanding, including the largest to-date (>6, 300 GBM cases) computational imaging study of GBM (Pati et al., 2022). Our analysis builds on the hypothesis that quantifying morphology patterns of GBM in WSI can yield biomarkers of prognostic relevance, while considering the lack of manual annotations and hence focusing on an unsupervised analysis. The computationally identified morphology patterns that drive the prognostic decisions of the resulting computational model in each patient's WSI were assessed and confirmed to be in agreement with the expert neuropathologist.

## 2 Materials and methods

### 2.1 Data

The proposed work is evaluated on the publicly available TCGA-GBM (Scarpace et al., 2016) and TCGA-LGG (Pedano et al., 2016) data collections, which are available through The Cancer Imaging Archive (TCIA) (Clark et al., 2013).

#### 2.1.1 Data reclassification

**Figure 1:**
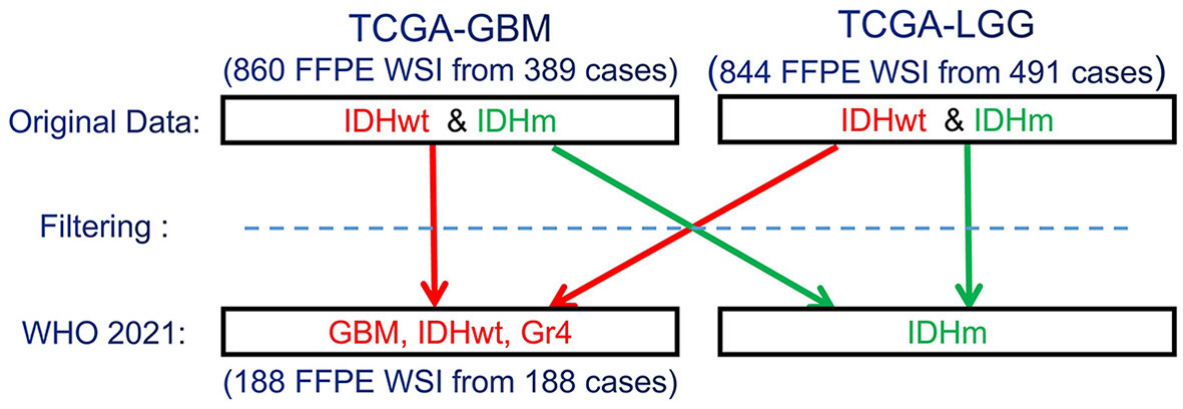
Schematic representation of the re-classification of the original TCGA-GBM and TCGA-LGG data collections, per WHO 2021 classification schema (Louis et al., 2021). “Filtering” describes the application of the additional specific inclusion criteria for the present study, such as cases with available overall survival (OS) information.

Since the CNS tumors of these data collections have been classified according to older WHO criteria, our expert neuropathologist (MPN) has reclassified these two complete data collections to identify and group together all GBM (IDH-wildtype, CNS WHO Gr.4) cases, according to the 2021 WHO classification of CNS tumors (Louis et al., 2021) (Figure 1). Based on the 2021 WHO CNS classification criteria, histologically low-grade astrocytomas from TCGA-LGG with molecular features of GBM are identified and also included in this study's cohort. Furthermore, few cases from TCGA-GBM have been excluded from being characterized GBM since their molecular profiles do not match the latest WHO-defined GBM entity of IDH-wildtype, CNS Gr.4. The molecular features assessed were *IDH* mutation, *TERT* promoter mutation, combined gain of chromosome 7 and loss of chromosome 10, codeletion of chromosomal arms 1p and 19q, pan-glioma RNA expression cluster, IDH-specific RNA expression cluster, pan-glioma DNA methylation cluster, supervised DNA methylation cluster, and random forest Sturm cluster.

#### 2.1.2 WSI selection and labeling

The TCGA-GBM and TCGA-LGG data collections include both formalin-fixed paraffin-embedded (FFPE) and frozen tissue section slides. Taking into consideration that frozen slides include hydration artifacts due to freezing, the complete analysis in this study was conducted on the available FFPE slides. Multiple FFPE Hematoxylin & Eosin (H&E)-stained WSIs were available per patient (ranging from 1 to 15), acquired from different parts of the resected tumor and scanned at a magnification level of either 20*X* or 40*X*. A single WSI from each available GBM patient case of the reclassified TCGA-GBM and TCGA-LGG collections is therefore utilized to maintain uniformity across the data assessed for each tumor. Furthermore, solid tumors may have a mixture of tissue architectures and structures, resulting in an inherent heterogeneity across the available multiple WSIs per patient. Hence, our expert neuropathologist (MPN) selected the exact WSI according to the apparent tumor proportion and appearance representative across all WSI of each patient, following processes conventionally involved in routine clinical practice. These cases are included irrespective of their scanning magnification level (20*X* or 40*X*).

A data-driven approach was initially used to detect paired cut-off values of the short- and long-survival patient groups. Specifically, these paired values were based on equal quartiles from the median OS, with the intention of mitigating class imbalance issues that could potentially affect negatively the donwstream model performance. The final cut-off values of ≤ 9 months and ≥ 13 months for short and long survivors, respectively, were determined following coordination with our clinical experts and while accounting for clinical significance and action. Patients with OS between these cut-off values were excluded, in order to avoid indeterminate cases near the cut-off boundaries. We excluded patients with survival exceeding 1,400 days (approximately 4 years—i.e., extreme outliers) and those with less than 15 days (around 2 weeks) to eliminate outliers. Further consideration of equal quartiles from median resulted in exclusion of 40 cases, leaving us with 188 cases for our study. This resulted in the short and long survivor classes having 94 cases each. 80% of the data (*n* = 152) is used for experimentation and model training, while 20% of the data (*n* = 36) is reserved as a hold-out unseen test set. The overall experimentation is performed in a 10-fold cross-validation configuration over the 80% of the complete data (*n* = 152) by further dividing them randomly and proportionally in training (80%), validation (10%), and testing (10%) sets. Then, once the experimentation concluded on the optimal parameter setting, the final model was trained on the 80% of the complete data (*n* = 152) and its quantitative performance evaluation was conducted on the hold-out unseen test set and reported in Section 3.

### 2.2 Approach

**Figure 2:**
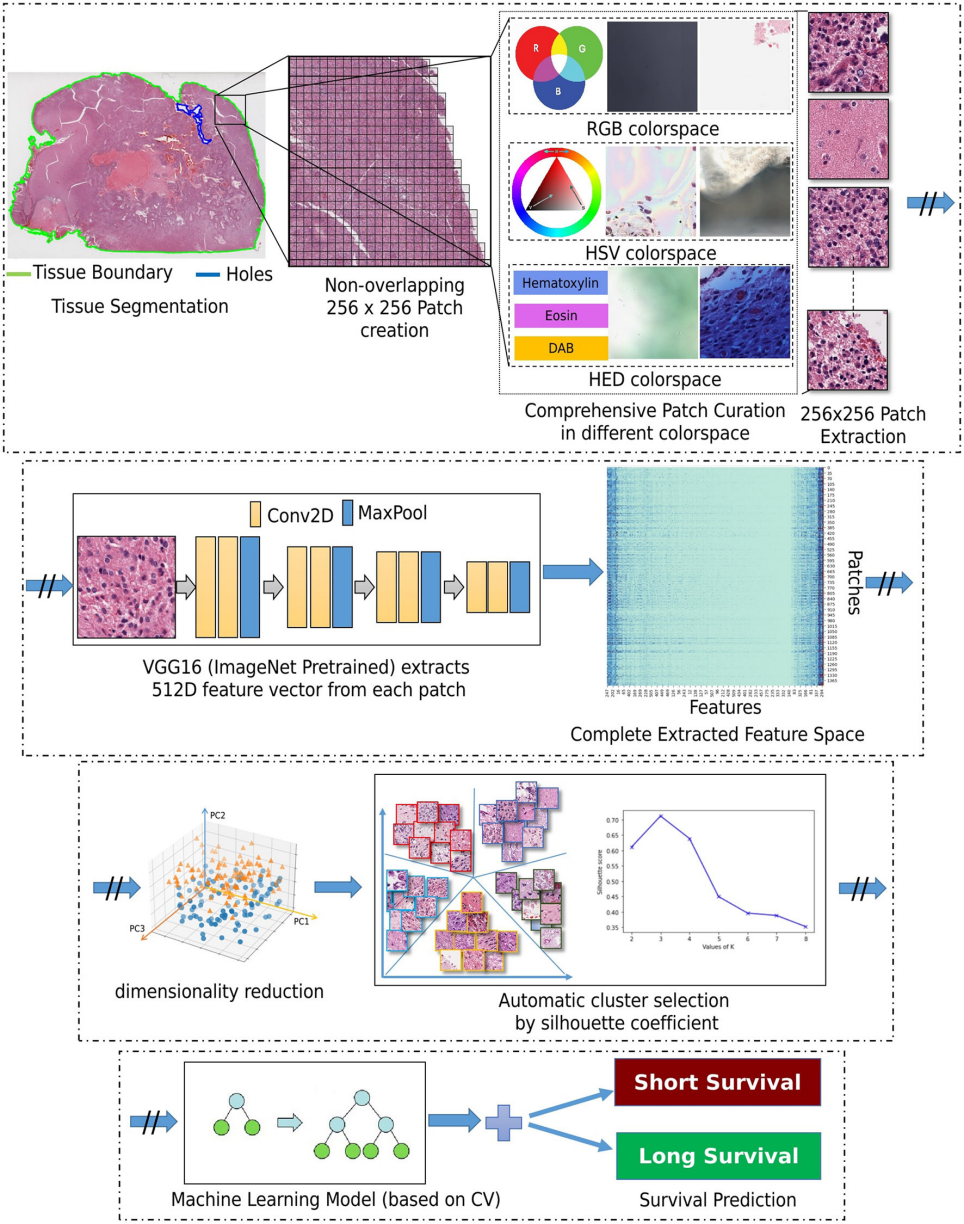
Schematic representation of the complete proposed methodological workflow.

Our approach is based on utilizing the entire WSI of each patient, and not by extracting a subset of patches from an annotated region (as typically done in the literature), nor by randomly selecting a fixed number of patches from a WSI to obtain the patient-level prognostic decision (Bejnordi et al., 2017; Mobadersany et al., 2018). This should allow for capturing the heterogeneous GBM morphology apparent in the complete WSI, in our attempt to capture all patterns of potential prognostic relevance. Figure 2 depicts the building blocks of our engineering approach, the complete details of which are given in the subsections below.

#### 2.2.1 Patch and feature extraction

Segmentation of the tissue section from the slide background is the first step in our approach. This is achieved by converting the second magnification level of the WSI pyramid into the Hue-Saturation-Value (HSV) colorspace and thresholding the WSI's saturation channel. Patches of size 256 × 256 are extracted from the segmented foreground tissue region at the 10*X* magnification level. Patches containing more than 60% background are discarded at this stage. Further comprehensive patch-level image curation excludes patches containing artifacts, such as glass reflections, pen markings, tissue folding, and black lines on the slide. The three steps of complete comprehensive curation are illustrated in Figure 2 (“Comprehensive patch curation”), with example artifact patches that each step removes.

Firstly, the algorithm discards the patches containing substantial white background or black regions if the percentage of such pixels within the Red-Green-Blue (RGB) colorspace is high (>60%). However, this step does not eliminate patches containing glass reflections, debris, or scanning artifacts. The algorithm eliminates such patches if the percentage of low-valued pixels in the saturation channel and high-valued pixels in the intensity channel of the HSV colorspace is >90%. Finally, the remaining patches with pen markings are removed by converting into a Hematoxylin-Eosin-DAB (HED) colorspace by stain deconvolution (Ruifrok et al., 2001) and checking if the associated intensity in the Eosin channel is >90%. All the selected thresholds are determined empirically by extensive experimentation on the given dataset to ensure that the algorithm only removes artifacts and preserves all tissue-occupied areas within the selected patches. Given another dataset, these thresholds could differ, and hence further experimentation is recommended for considering other data.

Following patch extraction, we use a convolutional neural network (CNN) for fully automated regional feature extraction from the WSI. Specifically, the CNN used was a VGG16 architecture pre-trained on ImageNet (Russakovsky et al., 2015) as it has been reported in the literature to capture related tissue patterns (Bychkov et al., 2018; Yao et al., 2019). The convolutional feature maps from the last block of the convolutional layer are extracted, and a global average pooling layer is applied on these feature maps to convert each 256 × 256 patch into a 512-dimensional feature vector. The benefit of representing the WSI into a matrix of features is lower computational cost and faster convergence during training.

#### 2.2.2 Dimensionality reduction

Dimensionality reduction of the extracted feature vectors is essential to avoid their potential overfitting to the assessed patient population. Specifically, principal component analysis (PCA) (Wold et al., 1987) is used to represent the extracted features in a lower dimensional space and improve our model's generalizability. PCA is a widely used approach for the dimensionality reduction of significant features by transforming a more extensive set of variables into a smaller one, which still contains most of the variability/information from the more extensive original collection. PCA reduces the number of dataset variables while preserving as much information as possible. As PCA is sensitive to the scale of input features, features are pre-processed by removing the mean and scaling to unit variance (i.e., z-scoring). As smaller feature vectors are easier to explore and analyze for machine learning algorithms, these reduced dimensional features are used for further processing.

#### 2.2.3 Unsupervised clustering

Subsequently, these feature vectors of lower dimensionality undergo a feature-based clustering approach [K-means (Hartigan and Wong, 1979)] to identify distinct groups/clusters of morphological patterns. Unlike supervised learning approaches, patch-level labels are unavailable in the unsupervised method of this study, making it a relatively more complex task to perform and evaluate. The objective is to discover patterns in the data following a data-driven approach, e.g., determining if there are any subgroups for which the collective characteristics of individual feature vectors have certain similarities. The K-means clustering requires a predefined number of clusters. However, since we want to let the data drive the decision about the (K) number of tissue phenotypes, we select the optimal number of clusters (K) by evaluating the goodness of clustering for different numbers of clusters (K = 2 to 10). The decision factor for the optimal number of clusters is estimated based on statistical analysis using both the rand index and the silhouette coefficient, quantifying cluster reproducibility and separability, respectively.

**Rand Index**

Rand index (RI) (Rand, 1971) is a measure to evaluate the reproducibility of clustering by finding the similarity of results between two different permutations. Similarity is computed by considering all sample pairs and counting pairs assigned in the same or different clusters. The RI can range from [0, 1]. The drawback of RI is that it assumes that the ground-truth cluster labels are available and uses them to compare the different permutations. As labels are not available in our case, we calculate the RI by comparing outputs of two different permutations with each other (performed with different random seeds) rather than a comparison with the ground truth.

**Silhouette coefficient and elbow method**

The silhouette coefficient is another approach used to determine the optimal number of clusters in data by evaluating the separability between clusters. It is a measure of how similar a feature is within its own cluster (cohesion) compared to the other clusters (separation) and ranges between [-1, 1]. According to the silhouette coefficient, a good clustering algorithm expects a high silhouette score, indicating slight within-cluster variance and significant between-cluster variance. The optimal number of clusters (K) is automated based on the elbow method, where the silhouette score is computed and plotted for a different number of clusters. The selection of K is at the point where the silhouette score drops suddenly.

Determining the number of PCA components (by retaining different amounts of variance), as well as the number of clusters, are hyperparameters and are decided by empirical experimentation. We first performed numerous experiments by varying PCs of the input CNN features and determined the optimal number of clusters (K) for further analysis. After K has been fixed, additional experiments are performed by retaining range of PCA components to analyze the effect of variance of PCA on classification accuracy. The K-means clustering algorithm was thus employed to identify the underlying morphological tissue phenotypes in WSI based on the extracted features.

#### 2.2.4 Classifier

After identifying the number of groups/clusters with unique morphological tissue patterns present in the WSIs, the tumor's spatial heterogeneity is captured by quantifying the proportion of each of the various identified morphological patterns within each patient's WSI. Such representation of a WSI conceptually provides a probability distribution of different tissue phenotypes and allows its description in a compact and meaningful way. Their association to prognostic stratification is explored by a decision tree (DT) classifier (Quinlan, 1986). The input features to the DT classifier were the k-means clustering output. Note that k-means was applied to the lower-dimensional feature vectors that were extracted from all WSI patches. Specifically, the probability distribution of each identified morphological pattern within a patient's WSI was used as an input feature. To optimize the DT's performance, we have conducted a grid search tuning of the associated hyperparameters, comprising depth, minimum sample leaves, minimum samples split, and criterion to measure the quality of a split. The previous steps such as patch extraction, feature extraction, PCA, and K-means clustering are carried out sequentially and independently for the training and the testing sets. Finally, the machine learning classifier is trained and quantitatively evaluated within each fold of the cross-validation schema. This sequential approach has been cautiously designed to prevent any potential information leakage and maintain the integrity of the reported performance evaluation.

## 3 Results

The k-means clustering algorithm (Hartigan and Wong, 1979) is inherently non-deterministic, and the initial selection of cluster centers can significantly influence the final clustering outcome. To correctly analyze the performance, clusters are manually and qualitatively inspected to determine whether the results are meaningful. To determine the optimal number of clusters quantitatively, we employ two key metrics: i) the rand index (Rathore et al., 2018a) and ii) the silhouette score (Shahapure and Nicholas, 2020), using the elbow method technique for different values (*K*∈{2, 10}). For rand index based metrics, we systematically vary the number of clusters (K) from 2 to 10 and execute K-means clustering on the reduced-dimensional CNN features for 1000 permutations, each with a distinct seed for initialization. To assess clustering stability and repeatability, we calculate the average rand index across these 1,000 permutations, together with different number of principal components (PCs) and the observed retained variance (Table 1). We observe that the Rand index is consistently close to 1 for all the performed experiments and permutations, despite the varying number of clusters and PCs. Such behavior is indicative of reproducible clustering (even after dimensionality reduction), and that even though GBM regions are histologically distinct they are also consistently captured across patients and grouped together by our unsupervised clustering algorithm.

**Table 1:** Rand index observed for different number of clusters and PCA components.

**Cluster**	**PCs = 10**	**PCs = 32**	**PCs = 64**
**Variance**	**35%**	**50%**	**60%**
2	0.9979	0.9981	0.9988
3	0.9895	0.9966	0.9971
4	0.9975	0.9988	0.9988
5	0.9969	0.9973	0.9975
6	0.9958	0.9970	0.9981
7	0.9947	0.9957	0.9971
8	0.9937	0.9895	0.9910
9	0.9897	0.9951	0.9979
10	0.9887	0.9988	0.9976

**Figure 3:**
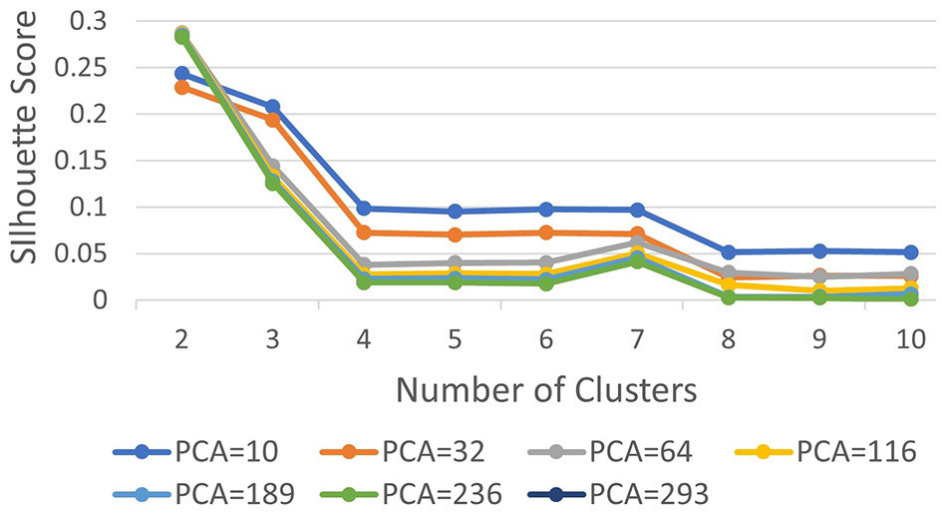
Silhouette scores with varying PCs.

**Figure 4:**
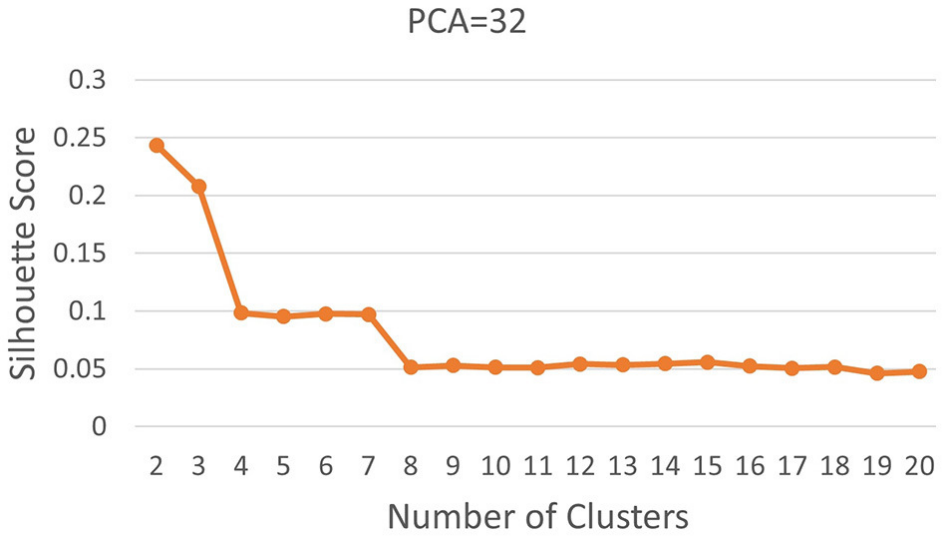
Silhouette scores for *K*∈[2, 20].

Subsequently, we computed silhouette scores across a range of number of clusters (*K*∈{2, 10}), while retaining the same number of PCs as in the rand index, and the obtained results are shown in Supplementary Table 1. These scores are further visualized in Figure 3, revealing an ‘elbow' point at *K* = 3 when 32 and 64 PCs are retained. In the case of 10 PCs, the initial elbow appears at *K* = 2, and as we proceed, another elbow emerges at *K* = 7, where plots with varying PCs counts converge. The “elbow” indicated at K=3, shows an inverse trend for 10 PCs compared to 32 and 64 PCs, whereas elbow patterns are consistent for K = 7. Opting for K=7 ensures consistency and captures the complex morphological patterns of GBM, toward enhancing clinical interpretability and introducing meaningful insights to a neuropathologist. The rationale behind limiting the explained variance by PCA to 60% was to reduce the dimensionality of the data and hence facilitate optimal cluster separability. In our exploration of retaining higher percentages of explained variance (70%, 80%, 85% and 90%) a high number of PCs were used that led to inferior cluster separability due to increased complexity. The exact values of the obtained silhouette scores, indicative of the cluster separability, are shown in Supplementary Table 1 and visually represented in Figure 3. Observation of Figure 3, indicates that higher percentage of variance leads to less abrupt/sharp elbows. Despite these variations, the stability and robustness of the selected number of clusters (K = 7) were consistent across different percentages of explained variance. Limiting PCA to 60% was considered a trade-off between variance and maintaining meaningful cluster separability. Additional analyses for assessing silhouette scores for clusters counts varying from 11 to 20 as represented in Figure 4 revealed no additional visible elbows. As a result, these observations strengthens the selection of *K* = 7 for our study.

The unsupervised clustering algorithm categorized each of these patches to a specific cluster based on its reduced dimensionality features where each cluster represents a unique morphology pattern. The patch distribution of various WSI in K clusters was not uniform, and some patients exhibited a dominance of a particular cluster, indicating a predominant morphological pattern within that WSI. This resulting distribution of clustering patterns are then used as inputs for the DT classifier. With the number of clusters determined, we conduct similar experiments using different numbers of PCs to achieve a retained variance ranging from 35% to 90% and the outcomes are detailed in Table 2, showcasing the fluctuating landscape of test accuracy, sensitivity, and specificity across different component counts. Sensitivity and specificity in this context indicate confidence in correctly predicting long and short survivors, respectively. Notably, as the number of components exceeds 50% of the retained variance, a decrease in sensitivity is observed while specificity remains stable. Additionally, this variation in the number of PCs was conducted as a sensitivity analysis to assess its impact on prognostic stratification while evaluating the performance of the machine learning classifiers.

**Table 2:** Results of classification on the independent hold-out test set with decision tree classifier for *K* = 7.

**#PCs (Variance)**	**Accuracy**	**Sensitivity**	**Specificity**
10 (35%)	0.7778	0.7778	0.7778
32 (50%)	0.8333	0.8333	0.8333
64 (60%)	0.6944	0.5556	0.8333
116 (70%)	0.6666	0.5556	0.7778
189 (80%)	0.7222	0.5	0.9444
236 (85%)	0.6667	0.5	0.8333
293 (90%)	0.6944	0.5	0.8889

The first column indicates number of PCs with the percentage of the retained variance mentioned within brackets.

The DT classifier reveals the nuanced impact of component selection on model performance, highlighting its ability to navigate the complex feature space generated by PCA and maintain balanced performance. Table 2 presents the results, demonstrating that the model achieves its highest accuracy of 83.33% when retaining 50% variance by reducing the dimensions to 32. This configuration also yields a sensitivity and specificity of 83.33%. However, sensitivity decreases as the number of components increases, illustrating the effectiveness of capturing meaningful patterns within the clustered data. Considering the observed drop in sensitivity with the Decision Tree (DT) model, we further explored Random Forest (RF) and XGBoost as alternative classifiers. Supplementary Table 2 includes detailed results of sensitivity, specificity, and accuracy for all three classifiers. After coordination with clinical experts, we aim to offer a method that correctly predicts either short or long-term survivors, to be clinically useful. Our decision to choose the DT over the other classifiers is based on consistently high specificity, i.e., high confidence in predicting long survivors, and hence a triaging mechanism. The highest specificity of 94.44% is observed when 80% of the input data variance was retained after dimensionality reduction. These findings underscore the critical role of selecting the appropriate number of components to optimize model performance, providing valuable insights for practitioners seeking to enhance real-world applications.

## 4 Discussion

In this study, we introduce an innovative engineering approach to distill holistic information from WSIs by grouping together similar phenotypical characteristics and prognostically stratify patients diagnosed with glioblastoma (GBM, IDH-wildtype, CNS WHO Gr.4). Our method harnesses the power of AI to enhance prognostic stratification by following an unsupervised computational paradigm as it does not require any manual annotations (or human intervention), and leverages deep learning for feature extraction and traditional machine learning approaches for the assessment and analysis of these features. The proposed analysis intended to delve deeper and further our understanding of this disease, shedding light on distinct morphological patterns that can aid in automated prognostic patient stratification. Prognostic stratification through WSIs represents a burgeoning field at the crossroads of medical imaging and machine learning. WSIs offer valuable insights into a patient's tissue sample, encompassing its histological features, morphology, physiology, and biology (Aeffner et al., 2019). A crucial step in our study was the reclassification of TCGA-GBM (Scarpace et al., 2016) and TCGA-LGG (Pedano et al., 2016) datasets, aligning them with the 2021 WHO CNS classification criteria (Louis et al., 2021). This alignment ensures the clinical relevance of our results, aligning them with the most up-to-date standards.

Even though more complex CNN architectures are available in the literature, compared to the VGG architecture (Simonyan and Zisserman, 2015) (selected here), they generate an even higher dimensional feature vector which imposes an inherent risk of overfitting due to their high dimensional feature space. The choice between a strong CNN and a simpler architecture depends on the specific goals of the task. A strong CNN might be more appropriate if the goal is to preserve a higher fidelity of the apparent WSI details. However, the critical caveat remains that there is no guarantee of the features learned by the encoder being optimal for the second-level machine learning block's goal, as these blocks are decoupled. Since we focus on retaining only key features for our downstream task (i.e., classification), a simpler architecture was considered sufficient. More complex architectures might capture fine-grained features that, when compressed, could potentially be lost or aggregated into less distinctive components. On the other hand, simpler architectures might extract more generalized features that could be more robust to compression and identifying underlying patterns when coupled with a clustering approach.

**Figure 5:**
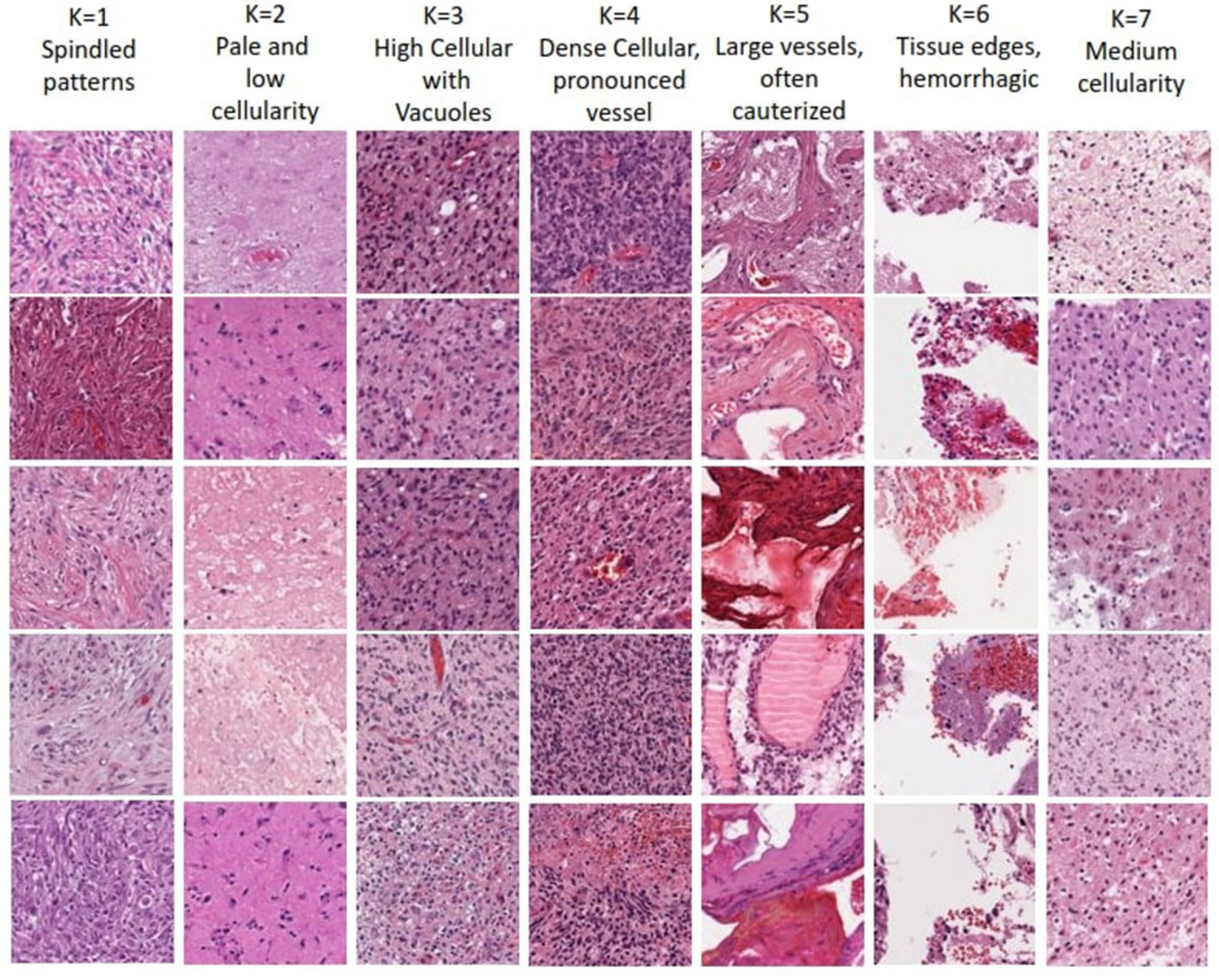
Visualization of morphological tissue patterns for seven clusters, with their interpretation.

The optimal number of K choosen according to results (i.e., K = 7) enables human expert uncover novel insights. Each of the seven clusters of distinct morphological patterns is given in Figure 5, where each cluster represents different characteristics of the tissue morphology. The first cluster contains atypical cells with spindled morphology. In contrast, the second cluster comprises subgroups with distinct attributes like infiltrative atypical cells with eosinophilic cytoplasm and pale, low cellularity, necrotic or macrophage-rich areas. High cellularity areas of the tumor with subsets of cells showing clearings, such as vacuoles or perinuclear haloes, are captured in the third cluster. The fourth cluster demonstrates the densest cellular areas with conspicuous vascularity. The fifth cluster captures tissue patches containing large-caliber vessels, often with signs of cauterization. Tissue edges, often with hemorrhage, are represented in the sixth cluster. The last cluster captures regions of glioma with intermediate cellularity between the second and third clusters. Neuropathological assessment in understanding these patterns, their proportions, and the relationship between them, can contribute to better characterizing the underlying tissue and potentially assist in improved diagnosing and classifying various conditions. Noting that the sequence of the presented clusters is irrelevant, analysis of these morphological patterns can contribute in identifying the vital histologic regions in the WSI that hold prognostic value.

**Figure 6:**
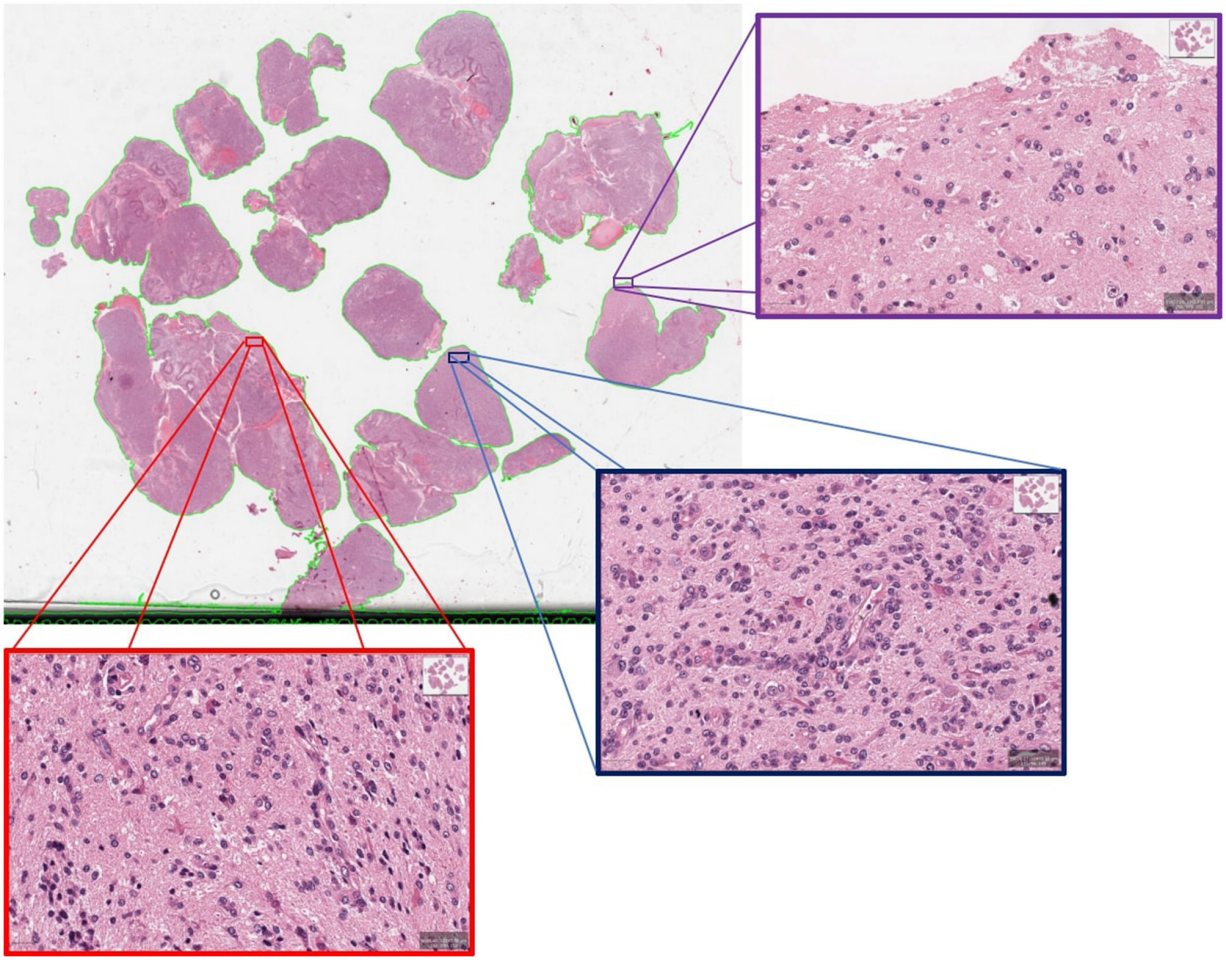
An example of misclassified case which is actually long survivor but predicted as short survivor.

We conducted a deeper assessment of the misclassified cases from the unseen hold out testing set, where specificity of 94.44% is observed when 80% PCA variance is retained. It revealed that this was the result of a single case misclassified as a short survivor, when the ground truth indicated this case as a long survivor, further highlighting the complexities of GBM prognosis. Visual neuropathologic assessment of the associated WSI (Figure 6) revealed high-grade histological features, including densely cellular infiltration, high mitotic activity, pseudopalisading necrosis, and abnormal vessels, that are consistent with an aggressive glioma with an unfavorable prognosis. Abundant pseudopalisading necrosis and abnormal vessels were present, including areas with microvascular proliferation. This case underscores the intricate nature of glioma prognosis and reveals where further model refinement is necessary. In addition, the conclusion may be reached that histological patterns are not always sufficient to predict outcomes (Burger and Green, 1987; Homma et al., 2006), given the known importance of molecular features of the tumor and the clinical details of each patient on clinical outcome. In any case, this misclassified case emphasizes the significance of collaborative expertise to refine and validate such predictions for accurate clinical decision-making. It also paves the way to future investigations aimed toward a deeper morphologic pattern analysis within each WSI.

A limiting aspect of our study lies in the inherent constraint of deriving our results based on a single (albeit the most representative) WSI per patient, especially in the context of the well-recognized heterogeneity of glioblastoma tumors. A single WSI per patient was selected based on the tissue selection process followed during true clinical practice, where a single tissue section is assessed according to the apparent tumor proportion and capturing representative appearance of the essential morphological features for accurate diagnosis. However, given that AI methods are data-driven and considering the generalization of our results in setting where other groups might not have the clinical capacity to select single WSI per patient according to this process, future research endeavors should focus on methodological development accommodating the utilization of multiple WSIs per patient. Such methodological innovation should account for the challenges associated with analyzing and integrating information from multiple WSIs, or even multiple magnification levels of the same WSI, offering a more comprehensive morphological profile of heterogeneous tissue samples associated with glioblastoma.

Further future directions include the exploration of multi-modal data, as well as incorporation of patient management and treatment course history that become crucial for differentiating mortality risk among patients with similar histology with greater precision and further subgrouping of tumors (Huang et al., 2020; Azam et al., 2022). Moreover, predictive models based on a single modality provide a limited view of disease heterogeneity and may not offer sufficient information to stratify patients (Soenksen et al., 2022). A holistic approach to patient prognosis should involve the integration of complementary information from heterogeneous data sources, including WSIs, molecular profiles, clinical data, and longitudinal radiology imaging (Lipkova et al., 2022). Qualitative analysis using only imaging data may prove insufficient, emphasizing the necessity of combining heterogeneous data streams for a comprehensive understanding of diseases, coupled with mechanisms of attention indicating drivers of algorithmic decisions (Baheti et al., 2023c). In the future, our focus will shift toward an integrated analysis of multiple modalities, aiming for a better understanding of the disease and prognosis. Additionally, a prospective evaluation on external multi-site data will be undertaken to ensure applicability in clinical workflows.

Intepretable machine learning plays a pivotal role in clinical settings, and our findings underscore the path forward in harnessing advanced models to create actionable, transparent, reproducible, and trustworthy clinical tools (Pati et al., 2023; Plass et al., 2023). Our interpretability analysis of diverse morphologic patterns within distinct histologic sub-regions of GBM may unveil further correlations with short and long survivors. This exploration offers a deeper insight into the intricate interplay between histologic features and clinical outcomes. Such insights can provide invaluable additional prognostic information to clinical neuropathologists during microscopic assessments, ultimately refining prognostication and potentially influencing clinical decision-making, thereby enhancing patient outcomes. Moreover, this newfound prognostic insight can guide the treating team toward promising avenues of research, furthering our understanding of GBM, improving their subgrouping and classification, potentially paving the way for innovative treatment approaches.

## Data availability statement

Publicly available datasets were analyzed in this study. This data can be found here: https://portal.gdc.cancer.gov/projects/TCGA-GBM; https://portal.gdc.cancer.gov/projects/TCGA-LGG.

## Author contributions

BB: Conceptualization, Data curation, Formal analysis, Funding acquisition, Investigation, Methodology, Software, Validation, Visualization, Writing – original draft, Writing – review & editing. SI: Data curation, Formal analysis, Investigation, Methodology, Software, Validation, Visualization, Writing – original draft, Writing – review & editing. MN: Data curation, Investigation, Supervision, Validation, Writing – review & editing. SB: Conceptualization, Funding acquisition, Investigation, Methodology, Resources, Supervision, Validation, Writing – review & editing.
