# Supplementary material for: Prognostic stratification of glioblastoma patients by unsupervised clustering of morphology patterns on whole slide images furthering our disease understanding
Source: Front Neurosci. 2024 May 20;18:1304191. doi: 10.3389/fnins.2024.1304191 (PMC11146603; doi:10.3389/fnins.2024.1304191)
Supplement: Supplementary file 1 [file Table_1.pdf]

SILHOUETTE SCORE ANALYSIS

This table shows values of silhouette score when number of clusters are varied from 2 to 10. The experiments are repeatedly performed to understand the effect of the number of principal components by varying percentage of retained variance on separability of clustering.

Table 1. Silhouette scores for different values of K from 2 to 10 by retaining different number of principal components.

Number of PC	PCs=10	PCs=32	PCs=64	PCs=116	PCs=189	PCs=236	PCs=293
Variance	35%	50%	60%	70%	80%	85%	85%
K=2	0.2899	0.2434	0.2287	0.2869	0.2859	0.2847	0.2828
K=3	0.1725	0.2077	0.1935	0.1444	0.1329	0.1280	0.1253
K=4	0.1698	0.0984	0.0725	0.0578	0.0474	0.0328	0.0287
K=5	0.1615	0.0951	0.0702	0.0598	0.0384	0.0234	0.0189
K=6	0.1644	0.0975	0.0724	0.0503	0.0280	0.0221	0.0175
K=7	0.1635	0.0969	0.071	0.0516	0.0503	0.0454	0.0412
K=8	0.1223	0.0513	0.0244	0.0194	0.0161	0.0033	0.0025
K=9	0.1141	0.0527	0.0261	0.0149	0.0101	0.0035	0.0022
K=10	0.1125	0.0513	0.0258	0.0181	0.0127	0.0063	0.0010

EXPERIMENTS WITH DIFFERENT MACHINE LEARNING CLASSIFIERS

This table presents the quantitative results of decision tree (DT), random forest (RF) and XGBoost (XGB) classifiers with the number of clusters (K) set to 7 on unseen test data in terms of accuracy, sensitivity and specificity. The results are organized based on the number of PCA components used for dimensionality reduction with percentage of retained variance mentioned in bracket and offer insights into the impact of varying numbers of PCA components on the performance of classifier.

Table 2. Quantitative results of various machine learning classifiers for K=7 and different number of principal components

Principal components	ML classifier	Test Accuracy	Test Sensitivity	Test Specificity
10 (35%)	DT	0.777777778	0.777777778	0.777777778
	RF	0.777777778	0.777777778	0.777777778
	XGB	0.5	0.333333333	0.666666667
32 (50%)	DT	0.833333333	0.833333333	0.833333333
	RF	0.694444444	0.555555556	0.833333333
	XGB	0.527777778	0.777777778	0.277777778
64 (60%)	DT	0.694444444	0.555555556	0.833333333
	RF	0.777777778	0.777777778	0.777777778
	XGB	0.694444444	0.944444444	0.444444444
116 (70%)	DT	0.666666667	0.555555556	0.777777778
	RF	0.722222222	0.777777778	0.666666667
	XGB	0.555555556	0.555555556	0.555555556
189 (80%)	DT	0.722222222	0.5	0.944444444
	RF	0.805555556	0.722222222	0.888888889
	XGB	0.722222222	0.666666667	0.777777778
236 (85%)	DT	0.666666667	0.5	0.833333333
	RF	0.694444444	0.722222222	0.666666667
	XGB	0.555555556	0.555555556	0.555555556
293 (90%)	DT	0.694444444	0.5	0.888888889
	RF	0.777777778	0.833333333	0.722222222
	XGB	0.722222222	0.666666667	0.777777778
